# Impact of the COVID-19 Pandemic on Objectively Measured Physical Activity and Sedentary Behavior Among Overweight Young Adults: Yearlong Longitudinal Analysis

**DOI:** 10.2196/28317

**Published:** 2021-11-24

**Authors:** Victoria Lawhun Costello, Guillaume Chevance, David Wing, Shadia J Mansour-Assi, Sydney Sharp, Natalie M Golaszewski, Elizabeth A Young, Michael Higgins, Anahi Ibarra, Britta Larsen, Job G Godino

**Affiliations:** 1 Herbert Wertheim School of Public Health and Human Longevity Science University of California, San Diego La Jolla, CA United States; 2 Center for Wireless and Population Health Systems University of California, San Diego La Jolla, CA United States; 3 Barcelona Institute for Global Health Barcelona Spain; 4 Exercise and Physical Activity Resource Center University of California, San Diego La Jolla, CA United States

**Keywords:** COVID-19, young adults, physical activity, sedentary behavior, activity monitor, public health, wearable, activity monitors, wrist worn, sedentary, lifestyle, pandemic

## Abstract

**Background:**

The COVID-19 pandemic has impacted multiple aspects of daily living, including behaviors associated with occupation, transportation, and health. It is unclear how these changes to daily living have impacted physical activity and sedentary behavior.

**Objective:**

In this study, we add to the growing body of research on the health impact of the COVID-19 pandemic by examining longitudinal changes in objectively measured daily physical activity and sedentary behavior among overweight or obese young adults participating in an ongoing weight loss trial in San Diego, California.

**Methods:**

Data were collected from 315 overweight or obese (BMI: range 25.0-39.9 kg/m^2^) participants aged from 18 to 35 years between November 1, 2019, and October 30, 2020, by using the Fitbit Charge 3 (Fitbit LLC). After conducting strict filtering to find valid data on consistent wear (>10 hours per day for ≥250 days), data from 97 participants were analyzed to detect multiple structural changes in time series of physical activity and sedentary behavior. An algorithm was designed to detect multiple structural changes. This allowed for the automatic identification and dating of these changes in linear regression models with CIs. The number of breakpoints in regression models was estimated by using the Bayesian information criterion and residual sum of squares; the optimal segmentation corresponded to the lowest Bayesian information criterion and residual sum of squares. To quantify the changes in each outcome during the periods identified, linear mixed effects analyses were conducted. In terms of key demographic characteristics, the 97 participants included in our analyses did not differ from the 210 participants who were excluded.

**Results:**

After the initiation of the shelter-in-place order in California on March 19, 2021, there were significant decreases in step counts (−2872 steps per day; 95% CI −2734 to −3010), light physical activity times (−41.9 minutes; 95% CI −39.5 to −44.3), and moderate-to-vigorous physical activity times (−12.2 minutes; 95% CI −10.6 to −13.8), as well as significant increases in sedentary behavior times (+52.8 minutes; 95% CI 47.0-58.5). The decreases were greater than the expected declines observed during winter holidays, and as of October 30, 2020, they have not returned to the levels observed prior to the initiation of shelter-in-place orders.

**Conclusions:**

Among overweight or obese young adults, physical activity times decreased and sedentary behavior times increased concurrently with the implementation of COVID-19 mitigation strategies. The health conditions associated with a sedentary lifestyle may be additional, unintended results of the COVID-19 pandemic.

## Introduction

Beginning in March 2020, many states in the United States implemented public health restrictions to reduce the transmission of SARS-CoV-2 and the incidence of COVID-19, including mandatory stay-at-home orders that have forced individuals to alter their family, work, education, and social routines. As a result, health behaviors are likely to have been affected [1,2]. These health behaviors include engaging in physical activity and minimizing sedentary behavior; their benefits across the life span have been well documented [2-5]. Importantly, increasing evidence suggests that these health behaviors are also associated with the risk of SARS-CoV-2 infection and the development of serious COVID-19 [2,4,5], thus making the understanding of how these health behaviors might have changed in response to COVID-19 mitigation strategies, including the introduction of the tiered reopening strategy at the end of August 2020 [6], a critical area of research.

The temporary closures of businesses and shifts from conducting in-person occupational and educational activities to conducting such activities in remote settings may have introduced new barriers to engaging in physical activity and reducing sedentary time, including limiting access to recreation spaces, increasing screen time [2,7], and altering sleep patterns [4]. Alternatively, the reductions in the time spent on other activities, such as commutes and social gatherings, may have increased the time available for engaging in physical activity. Therefore, it is unclear if changes in daily behavior are mitigating or exacerbating the separate, ongoing health crises of low physical activity and high sedentary behavior [2,5,8].

COVID-19 morbidity and mortality rates have been highest in patients who are older; are overweight or obese; and have associated comorbidities, such as type 2 diabetes mellitus and cardiovascular disease [9,10]. This is the same population that benefits the most from engaging in risk-reducing health behaviors; even a 2-week period of physical inactivity and increased sedentary behavior can measurably increase the risk of developing comorbidities [11,12]. Although older age is an important risk factor for COVID-19 complications, young adults are not impervious to serious COVID-19, especially young adults who are overweight or obese [13,14]. In recent decades, this demographic has experienced serious declines in physical activity and increases in sedentary behavior [4,15-17].

A growing number of studies show that physical activity decreased while sedentary behavior increased during the period of time that COVID-19 mitigation strategies were in effect [2,7,12,18,19]. These findings, while significant, are limited by cross-sectional study designs, the frequent use of convenience sampling with the limited characterization of study populations, and self-reported data of limited reliability and validity [2,14-16]. In this study, we add to the growing body of research on the health impact of the COVID-19 pandemic by examining longitudinal changes in objectively measured daily physical activity and sedentary behavior among young adults aged 18 to 37 years that have occurred prior to and throughout the ongoing pandemic (November 1, 2019, to October 30, 2020).

## Methods

### Participants and Setting

Our analyses used data from the Social and Mobile Approaches to Reduce Weight (SMART) 2.0 trial—an ongoing, 24-month (96 weeks), parallel-group randomized control trial that is being conducted in San Diego, California. The SMART 2.0 trial targets weight loss in overweight or obese young adults aged 18 to 35 years by using multiple modalities, including an activity monitor, wireless scale, and app; text messaging; social media platforms with social networking capabilities; and technology-mediated health coaching. The intervention content in the SMART 2.0 trial focuses primarily on self-regulatory mechanisms that promote health engagement in physical activity, diet, and sleep to achieve weight loss.

Participants were aged from 18 to 35 years old at enrollment; were overweight or obese (BMI: range 25.0-39.9 kg/m^2^); were affiliated with the University of California, San Diego; San Diego State University; or California State University, San Marcos; were Facebook users or were willing to begin using Facebook; and owned a smartphone. The exclusion criteria included having any comorbidities of obesity that require a clinical referral (ie, pseudotumor cerebri, sleep apnea, orthopedic problems, and type 2 diabetes), having psychiatric or medical conditions that prohibit compliance with the study protocol, or experiencing a cardiovascular event within 6 months of enrollment. Participants were also excluded if they were being treated for malignancy (other than nonmelanoma skin cancer), experienced an eating disorder, were planning to undergo weight loss surgery or engage in any other weight loss interventions or programs within 24 months of enrollment, or were pregnant or were actively planning to become pregnant within 24 months of enrollment.

All participants provided written informed consent prior to enrollment. Incentive payments of US $20, US $25, US $25, and US $30 were provided to participants at the 6-, 12-, 18-, and 24-month follow-up measurement visits, respectively. All participants’ study data were deidentified. The study procedures were approved by the University of California, San Diego, Institutional Review Board (approval number: 181862). The trial was sponsored by National Institutes of Health (grant NIH 5R01HL136769-01A1) and was registered with ClinicalTrials.gov (trial number: NCT03907462). The funder had no role in the research.

Demographic information was gathered through a self-reported survey at baseline, as shown in Table 1. All participants received weight loss goals that they were instructed to attempt to achieve throughout their participation in the study (ie, all participants were actively receiving the intervention). All participants were given the Fitbit Charge 3 (Fitbit LLC)—a wrist-worn activity monitor that measures physical activity, sleep, and heart function. They were instructed to wear the device daily. The device contains a triaxial accelerometer, optical heart rate monitor, altimeter, and vibration motor. The Fitbit uses a proprietary algorithm to determine activity levels, which are defined as vigorous, moderate, light, or sedentary [20]. Studies have shown that the Fitbit has an accuracy rating of 85.4% in distinguishing activity levels, specifically in distinguishing sedentary and light activity from moderate-to-vigorous activity [21]. Evidence has also shown that the Fitbit has acceptable levels of accuracy for measuring the number of daily steps taken [22,23]. The data from these devices were retrieved and aggregated by using the Fitabase software developed by Small Steps Labs LLC [24]—a third-party research platform designed to collect data from multiple Fitbit devices over time.

**Table 1:** Baseline characteristics of the participants in the Social and Mobile Approaches to Reduce Weight 2.0 trial who wore a Fitbit device for more than 250 days over the course of 1 year in California (N=97).

Characteristics			Participants, n (%)
**Sex**			
	Male	39 (40)	
	Female	58 (60)	
**Hispanic or Latino origin**			
	Yes	40 (41)	
	No	57 (59)	
**Race**			
	White or Caucasian	54 (56)	
	Black or African American	4 (4)	
	Asian	28 (29)	
	American Indian or Alaska Native	8 (8)	
	Native Hawaiian or Pacific Islander	1 (1)	
	Other	15 (16)	
**Highest level of education**			
	Less than high school	0 (0)	
	High school graduate	8 (8)	
	Some college or associate degree	44 (45)	
	College graduate or baccalaureate degree	21 (22)	
	Master's degree	24 (25)	
	Professional or vocational degree	0 (0)	
	Doctoral degree	0 (0)	
**Current relationship status**			
	Single or casually dating	44 (45)	
	In a committed relationship	33 (34)	
	Living in a marriage-like relationship	4 (4)	
	Married	15 (16)	
	Separated	0 (0)	
	Divorced	1 (1)	
**Income over the last 12 months (US $)**			
	<5000	28 (29)	
	5000-11,999	8 (8)	
	12,000-15,999	6 (6)	
	16,000-24,999	10 (10)	
	25,000-34,999	13 (13)	
	35,000-49,999	11 (11)	
	50,000-74,999	12 (12)	
	75,000-99,999	5 (5)	
	≥100,000	4 (4)	
**Number of children aged under 18 years that live in your home**			
	0	75 (77)	
	1	13 (13)	
	2	6 (6)	
	3	3 (3)	
**Number of adults that live in your home, including yourself**			
	1	15 (16)	
	2	33 (34)	
	3	19 (20)	
	4	18 (19)	
	5	4 (4)	
	6	7 (7)	
	10	1 (1)	
**What university are you affiliated with?**			
	University of California, San Diego	77 (79)	
	San Diego State University	6 (6)	
	California State University, San Marcos	14 (14)	
**What is your affiliation?**			
	Staff	32 (33)	
	Student	72 (74)	

The variables of interest included minutes of moderate-to-vigorous physical activity (MVPA; ≥3 metabolic equivalents of task [METs]), minutes of light physical activity (<3 METs), step counts (ambulation), and minutes of sedentary behavior (<1.5 METs). Data were acquired from Fitabase at the minute level for heart rate and at the daily level for all other metrics of interest (amount of time spent in sedentary, light, moderate, and vigorous activity; total number of steps; amount of time spent asleep). Heart rate data were analyzed, and aphysiologic data were removed (defined as unlikely heart rates of >200 beats per minute or <50 beats per minute). A new metric for the total number of minutes with a given heart rate per day was calculated and used as a proxy measure for wear time. This metric was further adjusted to create a variable for day wear. This was done by subtracting the number of sleep minutes calculated by the Fitbit’s proprietary algorithm for each calendar day (ie, the number of minutes from 12 AM to 11:59 PM that usually spans 2 sleep periods). The days that were included in the analysis were those in which participants achieved a day wear time of ≥600 minutes. The data were cleaned by using heart rate as an arbiter. Participant’s data were valid if they had a wear time of ≥600 minutes per day for at least 250 days in the year [25,26].

### Statistical Analysis

Changes in physical activity outcomes were first identified by using an algorithm that was designed to detect multiple structural changes in time series [27] and implemented within the R package *strucchange* (R Foundation for Statistical Computing). This method allows for the automatic identification and dating of structural changes in linear regression models with CIs. The optimal number of breakpoints in regression models was estimated by using the Bayesian information criterion and residual sum of squares; the optimal segmentation corresponded to the lowest Bayesian information criterion and residual sum of squares. To further quantify the changes in each outcome during the periods identified, linear mixed effects analyses were conducted. These analyses were implemented with the package *Lme4*, and posthoc comparisons among periods were conducted with the package *emmeans*. The mixed effects models included a random intercept, and posthoc comparisons were adjusted via the Bonferroni method. Finally, the four time series for steps, MVPA, light physical activity, and sedentary time were plotted with the package *ggplot2* by using a generalized additive model function. All statistical analyses were conducted by using R version 3.6.1 (R Foundation for Statistical Computing). The code and data used for the analyses are fully available on the Open Science Framework website [28]. The statistical significance of results was determined based on 2-tailed 95% CIs with predefined cutoffs. The data were stratified by date (ie, dates from November 1, 2019, to October 30, 2020). Descriptive statistics (proportions, means, and SDs) were used to define key demographic characteristics. Generalized additive models were conducted to analyze changes in each outcome variable over time.

## Results

### Summary of Participants

**Figure 1:**
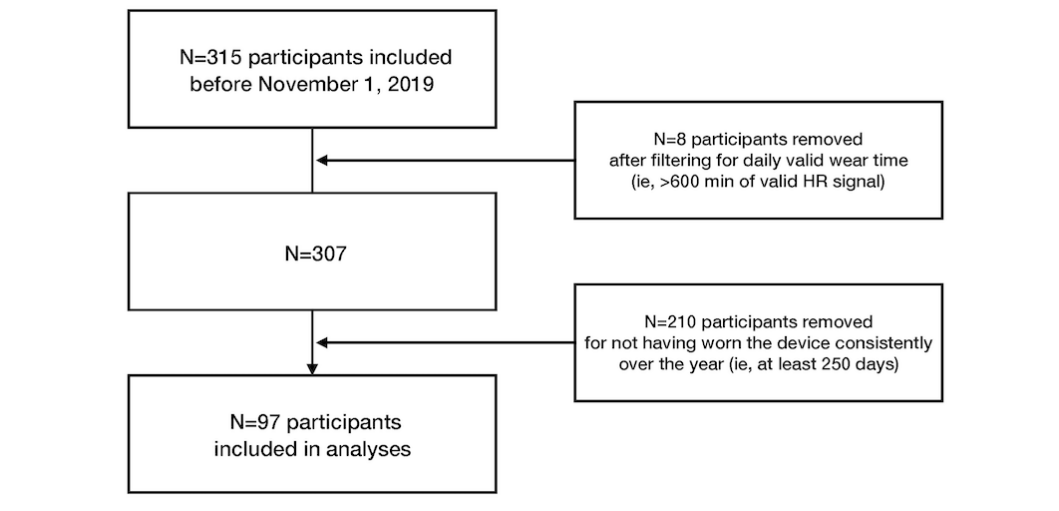
Flow diagram of participants in the Social and Mobile Approaches to Reduce Weight 2.0 trial who were included in the final analysis. HR: heart rate.

Data from a total of 315 participants were evaluated to determine their inclusion in this study. Among them, 8 participants were excluded after filtering for valid days based on our wear time criteria, as these 8 participants wore their devices for less 10 hours (600 minutes) per day. Afterward, 210 participants were excluded for having inconsistent wear times over the year. Specifically, these participants had less than 250 out of the valid 365 days’ (68.5%) worth of data. These rather strict cutoff criteria (ie, 250 days) were used in order to reflect an entire year of consistent wear time. A total of 97 participants had at least 250 valid days’ worth of monitoring data from November 1, 2019, to October 30, 2020, and were included in the analyses (Figure 1).

### Demographic Characteristics

Table 1 shows the unadjusted study sample characteristics of the 97 participants included in the final analysis. The average age of the study population was 26.5 years (SD 8.5 years). Further, 41% (40/97) of participants identified as Latino or Hispanic, 44% were people of color (43/97), and 60% (58/97) identified as female. On average, participants had 45 minutes (SD 15 minutes) of MVPA, 6200 steps (SD 2000 steps), 367 minutes (SD 67 minutes) of sleep, and 1020 minutes (SD 108 minutes) of sedentary time per day throughout the analysis. The 97 participants included in the analyses did not differ from the 210 participants who were excluded in terms of the key demographic characteristics reported.

### Changes in Physical Activity and Sedentary Behavior

**Figure 2:**
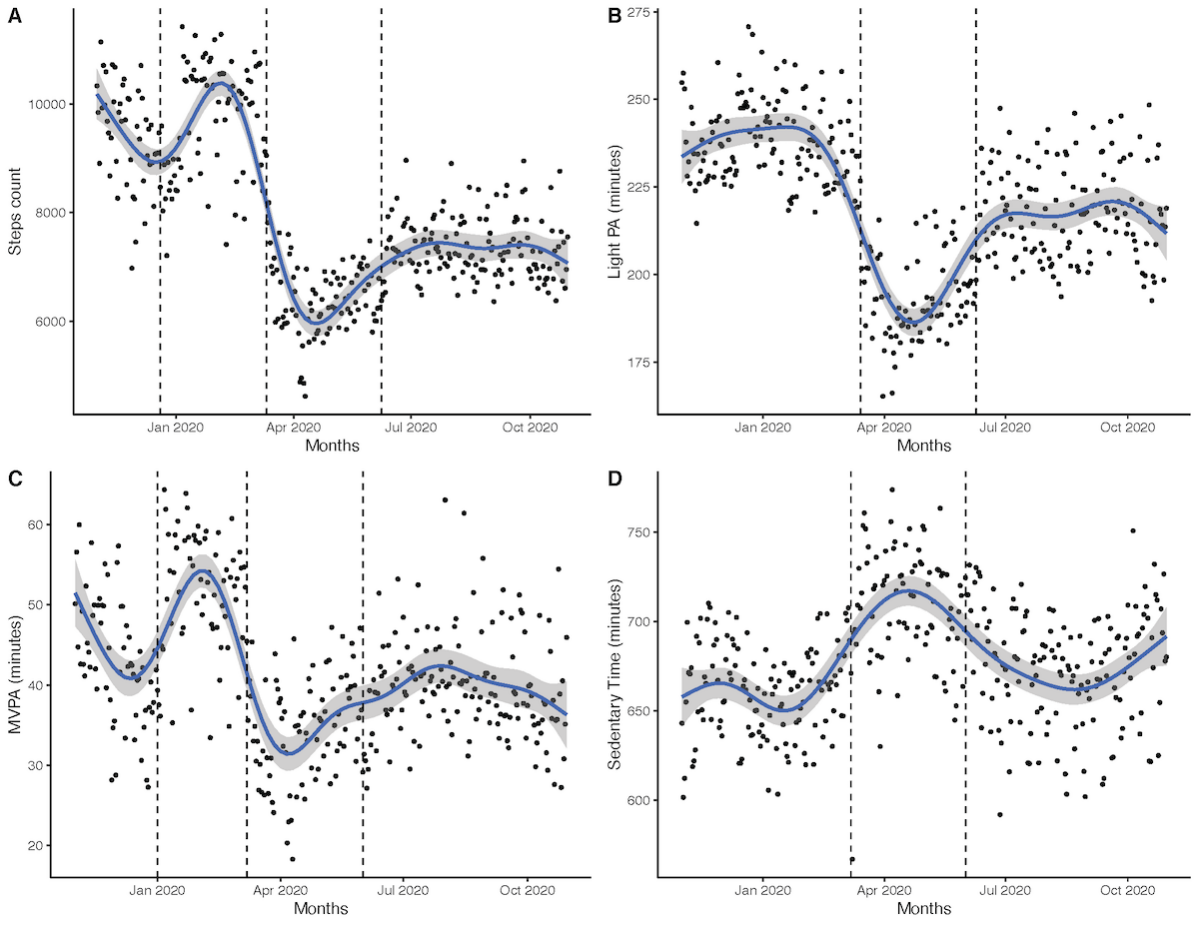
Changes in the mean (A) step count, (B) light PA time, (C) MVPA time, and (D) sedentary time. Three breaks in the time series are highlighted at the end of December 2019, mid-March 2020, and July 2020 among participants of the Social and Mobile Approaches to Reduce Weight 2.0 trial who wore a Fitbit (Fitbit LLC) device for more than 250 days over the course of 1 year in California. MVPA: moderate-to-vigorous physical activity; PA: physical activity.

Many of the breaks in activity occurred concurrently with the implementation of pandemic mitigation strategies in San Diego County, including the closing of schools, gyms, recreation spaces, parks, beaches, and other businesses in mid-March 2020; the reopening of outdoor spaces at the end of April 2020 and in early June 2020; and the introduction of the tiered reopening strategy at the end of August 2020. As shown in Figure 2, there was a marked decrease in step counts, light physical activity times, and MVPA times, as well as an increase in sedentary times, in March 2020 when compared to those in the prior months. For step count, the structural break detection algorithm indicated that 3 breaks in the time series occurred—one between December 20, 2019, and January 20, 2020; one between March 11 and March 13, 2020; and another between June 8 and June 18, 2020. Additionally, 3 structural breaks were also detected for MVPA. These occurred between January 1 and 14, 2020; between March 7 and 10, 2020; and between June 13 and 18, 2020. Further, 2 structural breaks were detected for light physical activity. These occurred between March 14 and 16, 2020, and between June 9 and 14, 2020. For sedentary behavior, 2 breaks occurred—one between March 7 and 14, 2020, and another between June 1 and June 16, 2020. Taken together, these results suggest that a significant increase in step counts and MVPA times occurred at the beginning of the year after the holiday season, a net decrease in physical activity outcomes occurred in mid-March (between March 10 and 16), and a new increase in physical activity times and decrease in sedentary behavior times occurred in the first 2 weeks of June. Multimedia Appendix 1 provides further information on these models.

**Figure 3:**
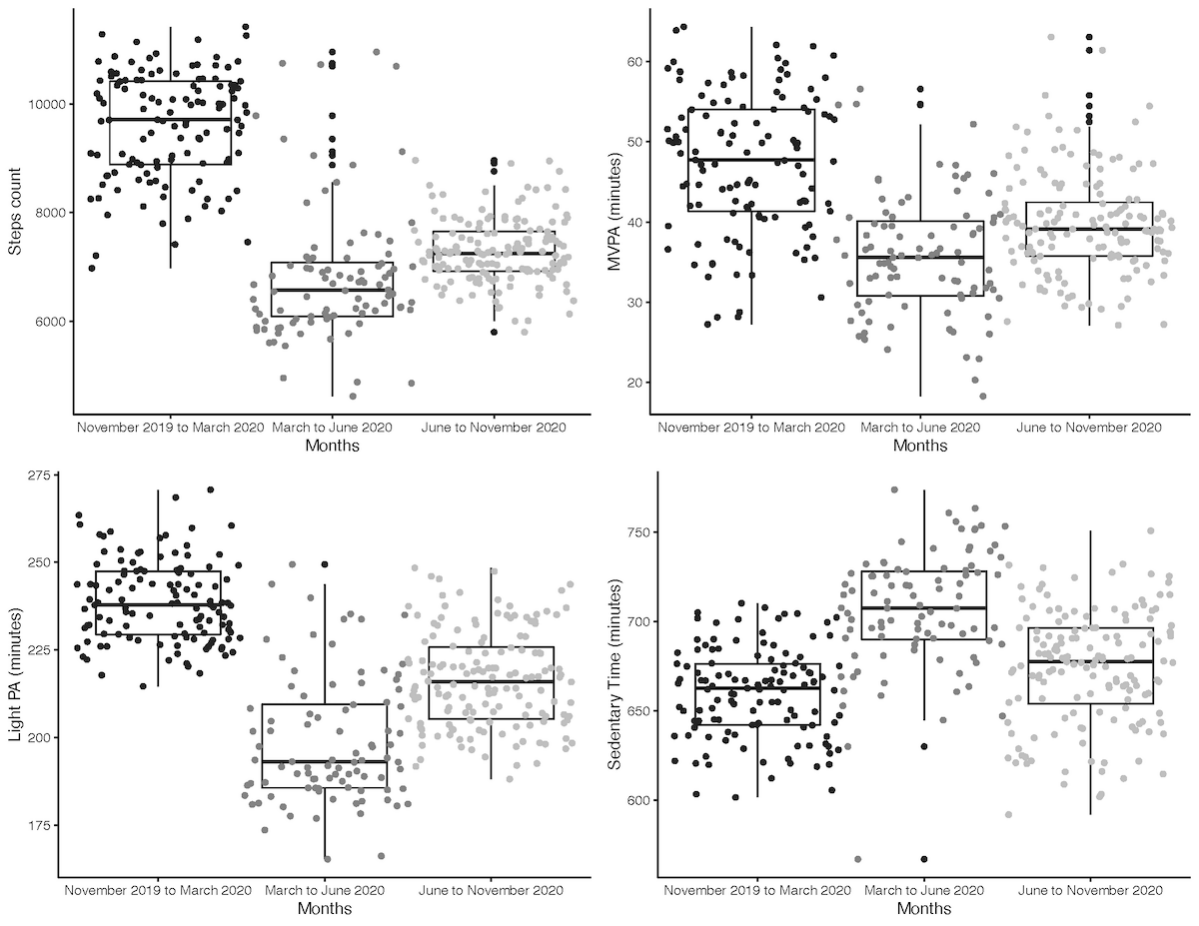
The results from linear mixed effect models confirmed significant differences in (A) step counts, (B) MVPA times, (C) light PA times, and (D) sedentary times across the three periods (November 2019 to the end of February 2020, the beginning of March to the end of May, and the beginning of June to October 2020) among participants of the Social and Mobile Approaches to Reduce Weight 2.0 trial who wore a Fitbit (Fitbit LLC) device for more than 250 days over the course of 1 year in California. MVPA: moderate-to-vigorous physical activity; PA: physical activity.

The results from linear mixed effect models confirmed significant differences in each outcome among the following three periods: November 2019 to end of February 2020 (period 1), the beginning of March 2020 to the end of May 2020 (period 2), and the beginning of June 2020 to October 2020 (period 3; Figure 3).

The average daily numbers of steps per day were 9641 (SE 251; period 1), 6769 (SE 253; period 2) and 7299 (SE 251; period 3) for the three time periods. A significant drop of 2872 steps per day was observed between periods 1 and 2 (95% CI 2734-3010), a significant increase in step count occurred between periods 2 and 3 (+529 steps per day; 95% CI 396-663), and the average number of steps in period 3 was still significantly lower than that in period 1 (−2343 steps per day; 95% CI −2223 to −2463).

Similar patterns of results were observed for the other outcomes. The average number of minutes of MVPA per day was 47.6 minutes (SE 2 minutes) in period 1, 35.4 minutes (SE 2 minutes) in period 2 (−12.2 minutes; 95% CI −10.6 to −13.8), and 39.9 minutes (SE 2 minutes) in period 3 (+4.5 minutes compared to minutes in period 2; 95% CI 2.9-6).

The average number of minutes of light physical activity per day was 239 minutes (SE 5 minutes) in period 1, 197 minutes (SE 5 minutes) in period 2 (−41.9 minutes; 95% CI −39.5 to −44.3), and 216 minutes (SE 5 minutes) in period 3 (+19.1 minutes compared to minutes in period 2; 95% CI 16.7-21.4).

The average number of minutes of sedentary behavior per day was 659 minutes (SE 10 minutes) in period 1, 712 minutes (SE 10 minutes) in period 2 (+52.8 minutes; 95% CI 47-58.5), and 678 minutes (SE 10 minutes) in period 3 (−34 minutes compared to minutes in period 2; 95% CI −28.4 to −39.6).

**Figure 4:**
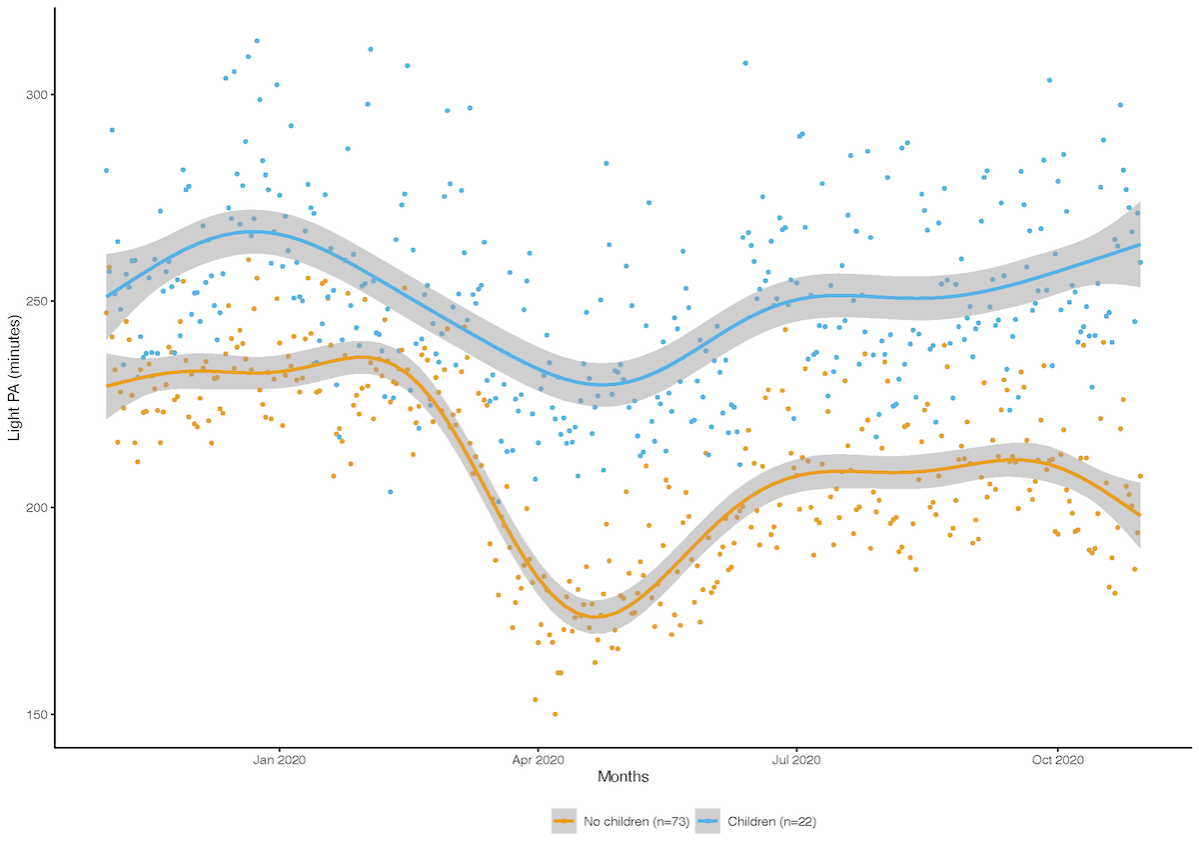
Changes in the mean light PA times of participants of the Social and Mobile Approaches to Reduce Weight 2.0 trial who wore a Fitbit (Fitbit LLC) device for more than 250 days over the course of 1 year in California. The declines in light physical activity times among participants with children (n=22) were lower than those among participants without children (n=73). PA: physical activity.

The times series highlighted that these patterns of changes were relatively similar across subgroup comparisons, including those between males and females; between single and committed participants; between participants with annual incomes of below and above US $25,000; and between participants with and without children (Multimedia Appendix 2), with one exception. The declines in light physical activity times among participants with children (n=22) were lower than those among participants without children (n=73; Figure 4). Light physical activity was significantly different between these two groups during period 2 (−45.4 minutes for the participants without children; 95% CI −79.98 to −10.90) as well as during period 3 (−43.63 minutes for the participants without children; 95% CI −78.01 to −9.25).

## Discussion

### Principal Findings

In our study, we analyzed a complete year’s worth of objective, high-resolution data to assess the impact that mitigation strategies associated with the COVID-19 pandemic have on physical activity and sedentary behavior in young adults. We observed that after the initiation of the shelter-in-place order in California, there were significant decreases in step counts, light physical activity, and MVPA, as well as significant increases in sedentary behavior. The decreases were greater than the expected declines observed during winter holidays, and they have not returned to the levels observed prior to the initiation of shelter-in-place orders. The length of the time series used in this study provides valuable insight into the effects of COVID-19 mitigation strategies. Specifically, strategies that include reducing access to recreation spaces and gyms and shifting to remote, web-based work rather than commuting to work or school are likely causes of the reductions in physical activity and increases in sedentary behavior observed within our study population [2-4,29]. These mitigation strategies, while important for reducing the spread of infectious disease, may further exacerbate the ongoing and separate health crises of low physical activity and high sedentary behavior that are present in the young adult population.

Our findings are in line with the findings from the existing literature to date, which has relied on self-report surveys and vary in length, ranging from 24-hour recalls [30] to 6-month self-recall physical activity reports [2,31]. Decreases in step counts, MVPA, and light physical activity and increases in sedentary behavior have been reported [17,19,30]. There have also been reports of increased activity in several populations; however, these patterns are unequal, as they depended on access to spaces for physical activity as well as whether these populations met the recommended level of physical activity prior to the initiation of pandemic mitigation strategies [32,33]. Most data have found that over 30% of adults have reported declines in physical activity in response to strict lockdown ordinances [2,34]. Our results expand the research in this area by revealing the magnitudes of the declines in physical activity and increases in sedentary behavior throughout key moments within the pandemic. This analysis captures the scope of the predicted declines in physical activity (November 2019 through the end of February 2020) resulting from holidays impacting work, family, education, and social routines and the daily behaviors associated with these declines. These were analyzed in relation to the initial mitigation strategies that were implemented from the beginning of March through May 2020. Such strategies included the March 19, 2020, stay-at-home order, which resulted in the closing of gyms, beaches, parks, and recreation spaces, and the subsequent opening of beaches and parks on April 27, 2020. We also identified a period of increased activity, which occurred from June 2020 through October 2020. This coincided with the June 12, 2020, reopening of some gyms and businesses and the tiered reopening system that took effect on August 31, 2020, during which physical activity and sedentary behavior levels were still lower than those from before the pandemic.

Our results showed that these trends were consistent across gender, partner status, income, and whether participants had children, underscoring the strong effect of COVID-19 mitigation strategies across all subgroups. The pronounced decreases in physical activity highlight a need for further investigation into the reasons for these declines; while many local restrictions resulted in the closures of gyms and other recreational areas, even the strictest stay-at-home orders all allowed for outdoor physical activity, and many gyms continued to conduct operations and classes outdoors. Additionally, considerable reductions in commuting time resulting from work and school being conducted remotely provided many individuals with increased leisure time. It is likely, then, that reductions in activity were due to changes in lifestyle activity associated with occupational and transportation-related physical activity and due to individuals being unable or unwilling to adapt to routines that accommodate for COVID-19–related restrictions, though more research on this is needed.

Our findings highlight a vast need for interventions that focus on increasing physical activity and decreasing sedentary behavior in young adults and the need for these interventions to highlight problem-solving and adaptations to changing conditions. Importantly, increasing evidence suggests that physical activity can reduce the risk of SARS-CoV-2 infection and the development of serious COVID-19 [2,4,5]. Given these risks, the increases in screen time associated with remote work and school, and the significant deleterious effects that the COVID-19 pandemic has on mental health, maintaining and increasing physical activity are key strategies for reducing some of the greatest mental and physical health risks associated with the COVID-19 pandemic. The World Health Organization has predicted that more global health emergencies will occur in the future [35]; the success of future health behavior interventions may depend on their ability to adapt to changing conditions at the global and individual levels.

The strengths of this study include the use of objective data from an existing cohort that has been longitudinally observed over the course of an entire year. This provided us with the ability to segment and examine expected declines in physical activity due to holidays and unexpected declines resulting from COVID-19 mitigation efforts. The limitations include the use of a sample that was recruited entirely from San Diego, California. This makes our findings potentially less generalizable to all young adults in other regions. The participants were also a part of an ongoing weight loss study and were seeking to lose weight, which may further limit the generalizability of our results to those attempting to lose weight and those who are motivated to use a Fitbit to support their weight loss. Additionally, due to the conservative cutoff criteria used for valid days and consistent wear times, our analysis included a smaller sample size compared to the original study’s larger sample size.

### Conclusion

In summary, our findings support the observation that health behaviors in young adults have been significantly impacted by COVID-19 mitigation strategies. The strategies used to mitigate and control the spread of SARS-CoV-2, while important, have unintended consequences that may continue to become increasingly apparent in the young adult population following the COVID-19 pandemic. As the pandemic continues, this population will be faced with a different lifestyle from the one before the pandemic—one in which the need for improved health behaviors should be emphasized beyond the pandemic and as pandemic fatigue [27] continues to negatively impact lifestyle decisions. Future interventions aimed at young adults should include varied options for physical activity, including options for conducting physical activities at home and in the community, as well as strategies for decreasing sedentary behavior, as most activities of daily living now occur in a home environment.

## Appendix

Multimedia Appendix 1 Structural break detection results.

Multimedia Appendix 2 Time series data.

## Abbreviations

MET: metabolic equivalent of task
MVPA: moderate-to-vigorous physical activity
SMART: Social and Mobile Approaches to Reduce Weight
