# Supplementary material for: Impact of the COVID-19 Pandemic on Objectively Measured Physical Activity and Sedentary Behavior Among Overweight Young Adults: Yearlong Longitudinal Analysis
Source: JMIR Public Health Surveill. 2021 Nov 24;7(11):e28317. doi: 10.2196/28317 (PMC8614391; doi:10.2196/28317)

Structural break detection for steps

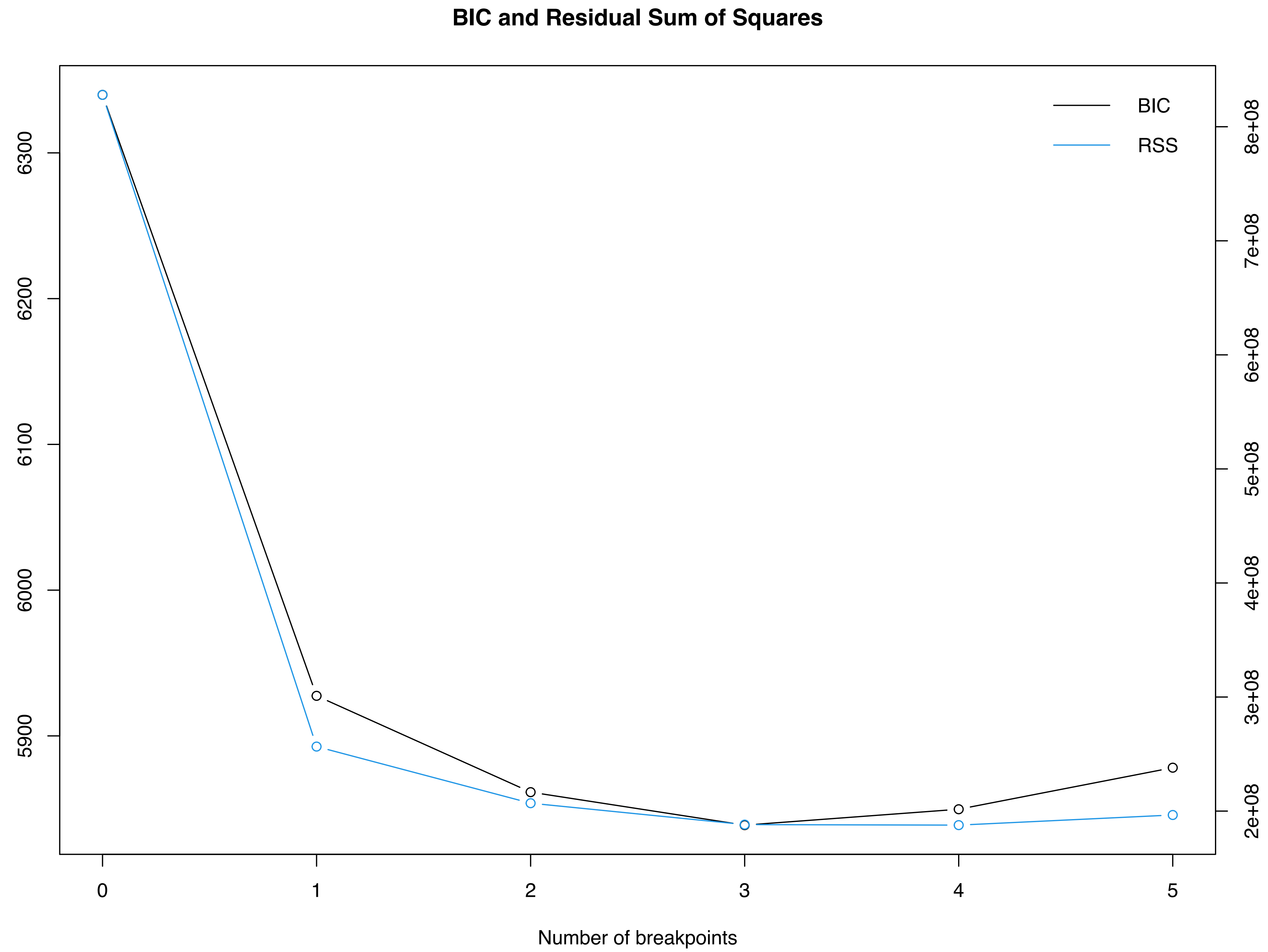

Structural break detection for steps

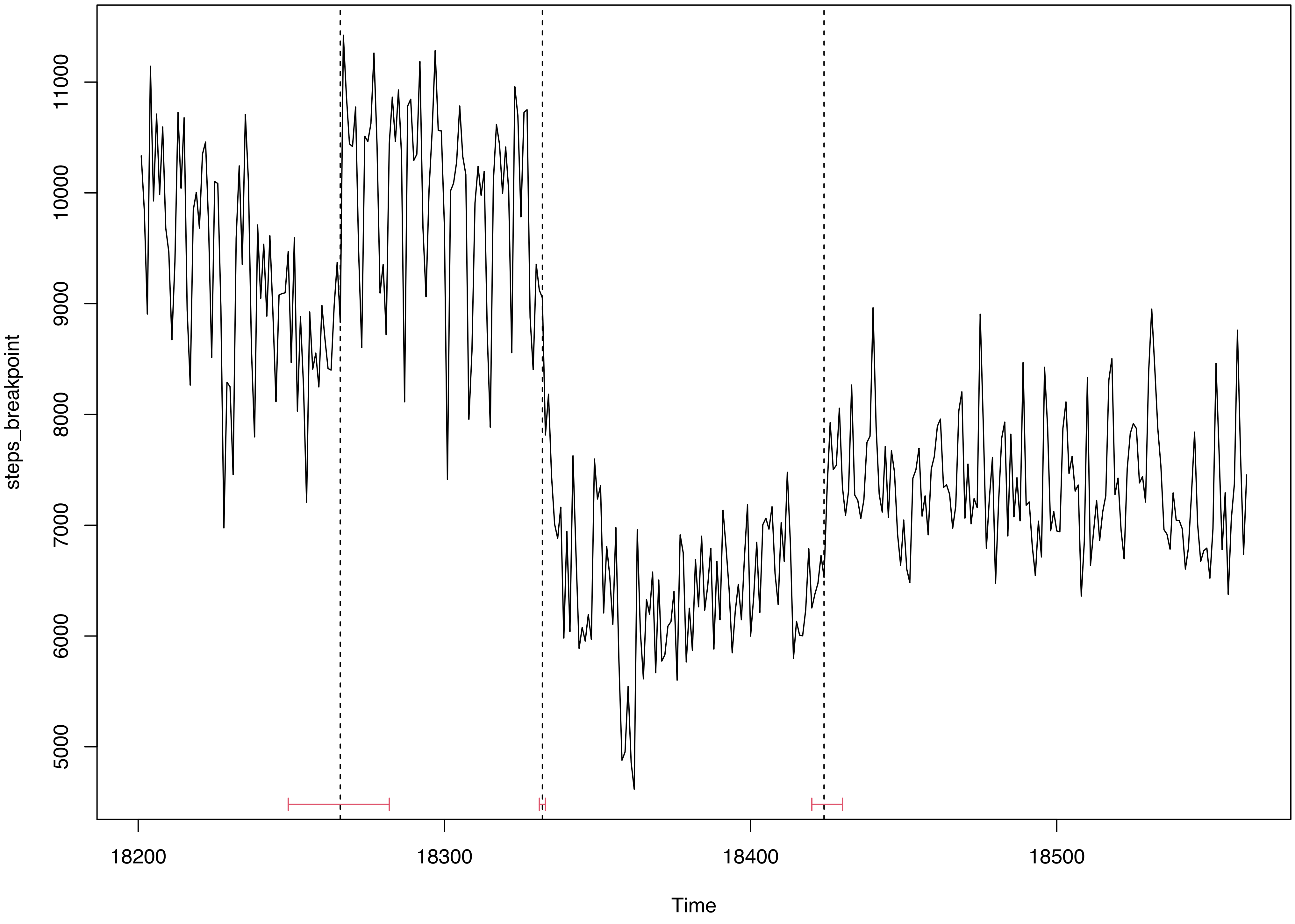

Structural break detection for light PA

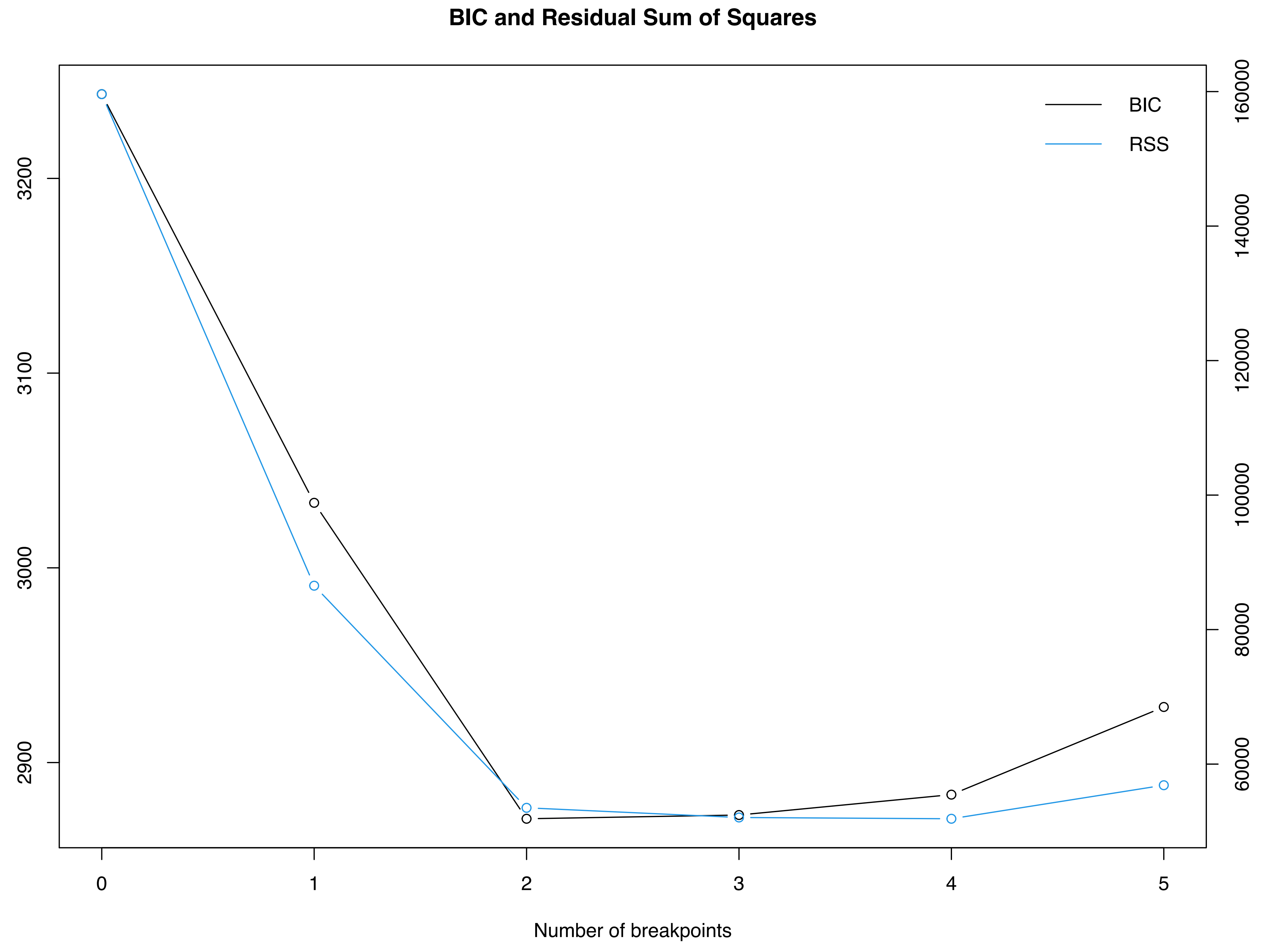

Structural break detection for light PA

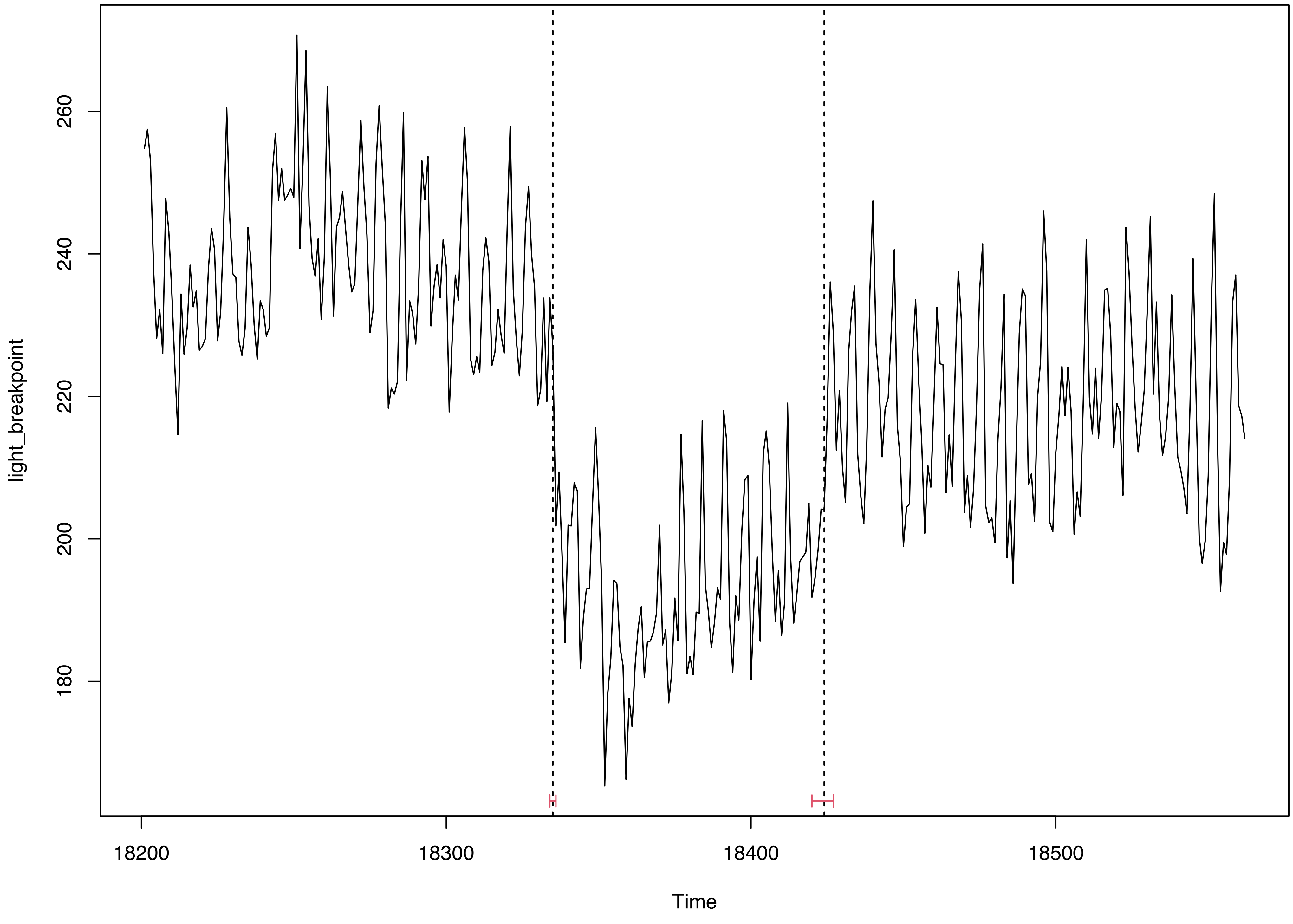

Structural break detection for MVPA

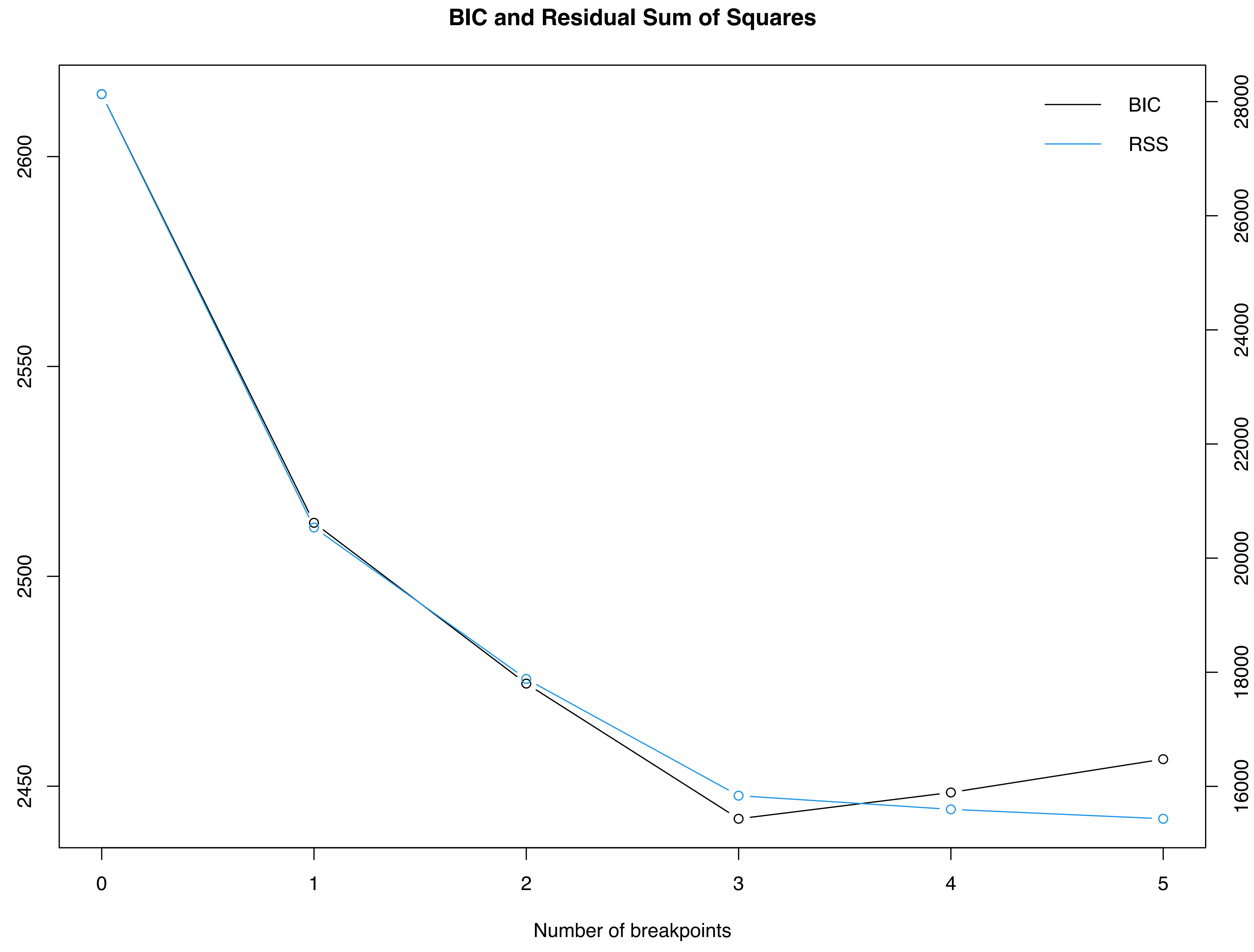

Structural break detection for MVPA

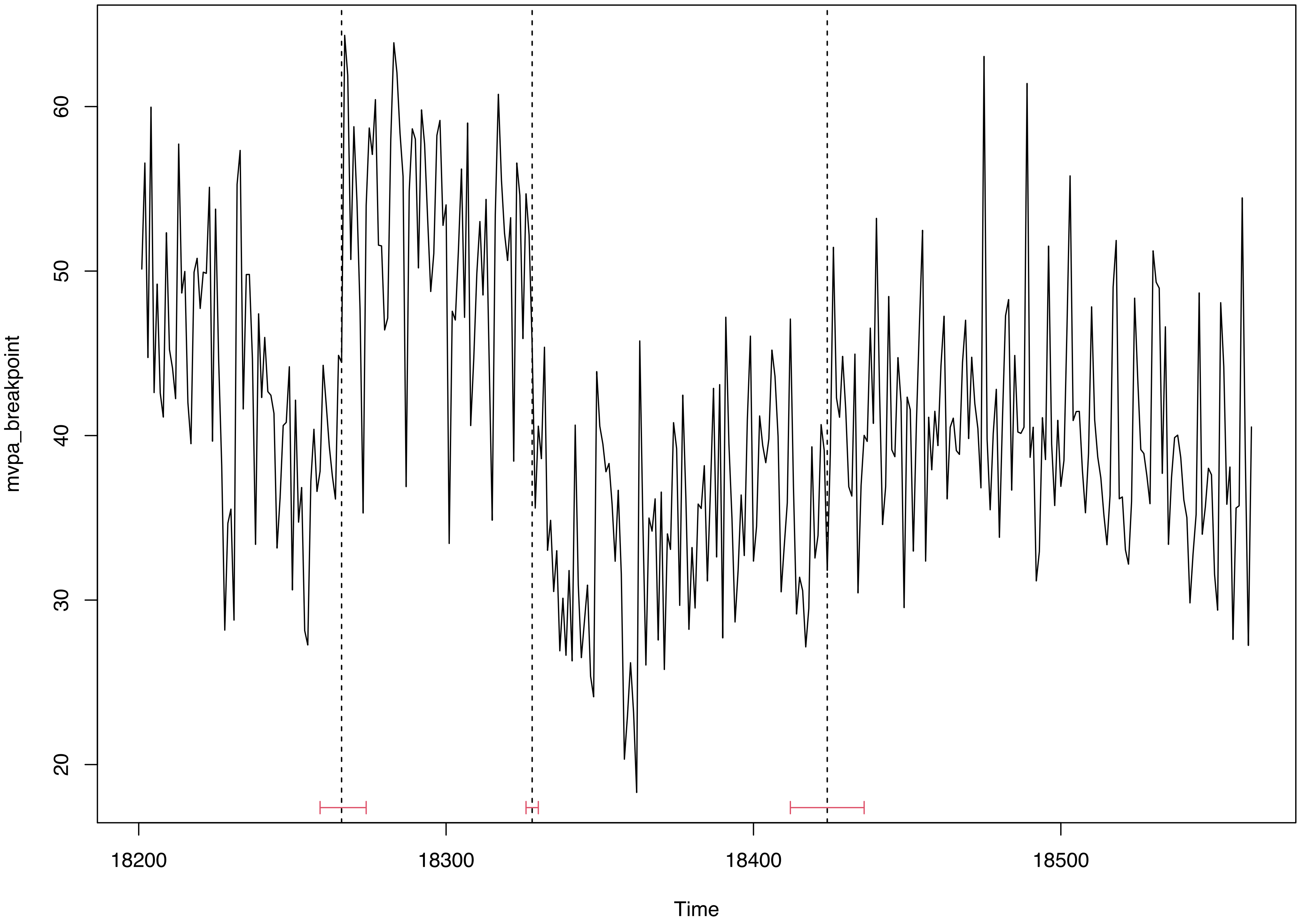

Structural break detection for sedentary behavior

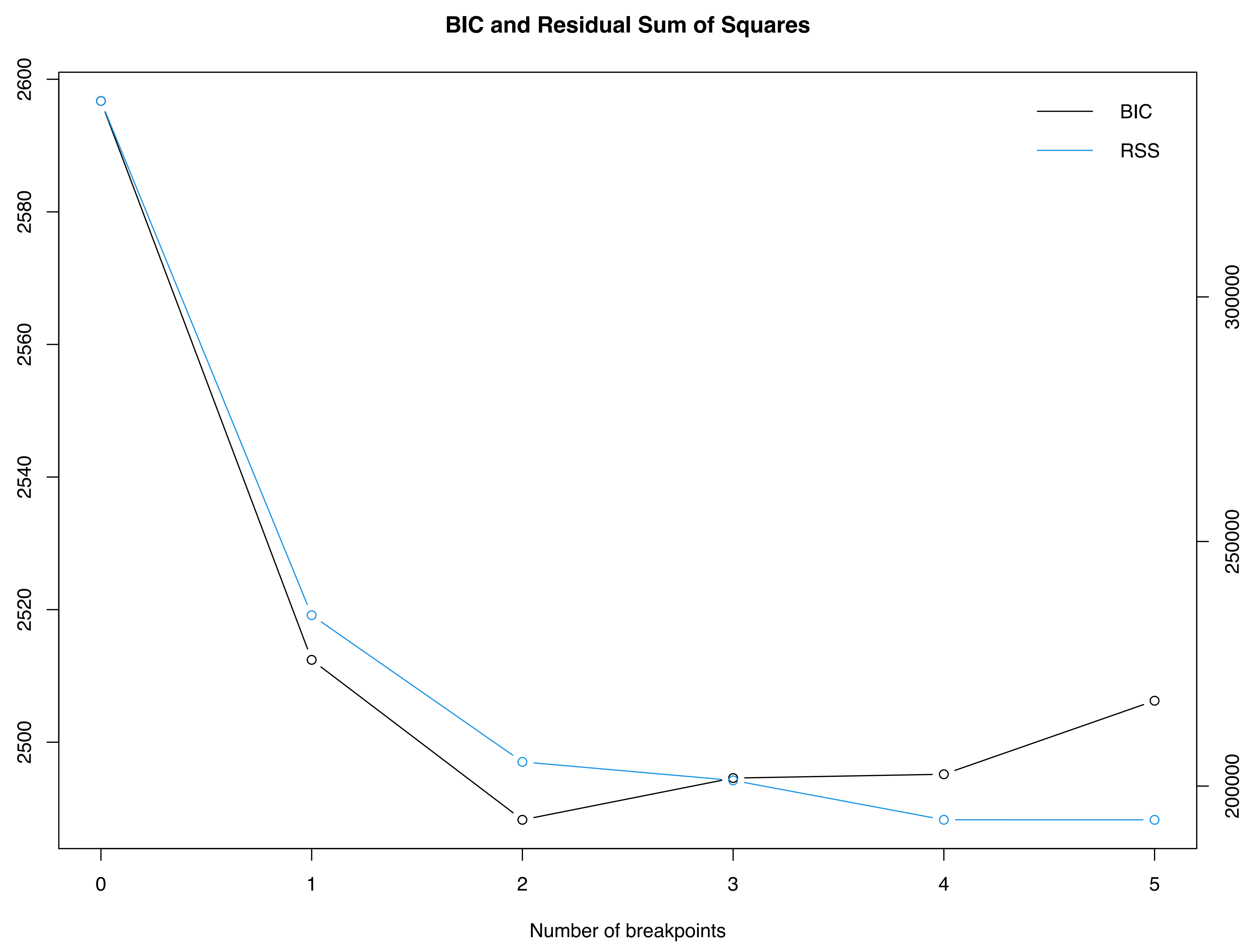

Structural break detection for sedentary behavior

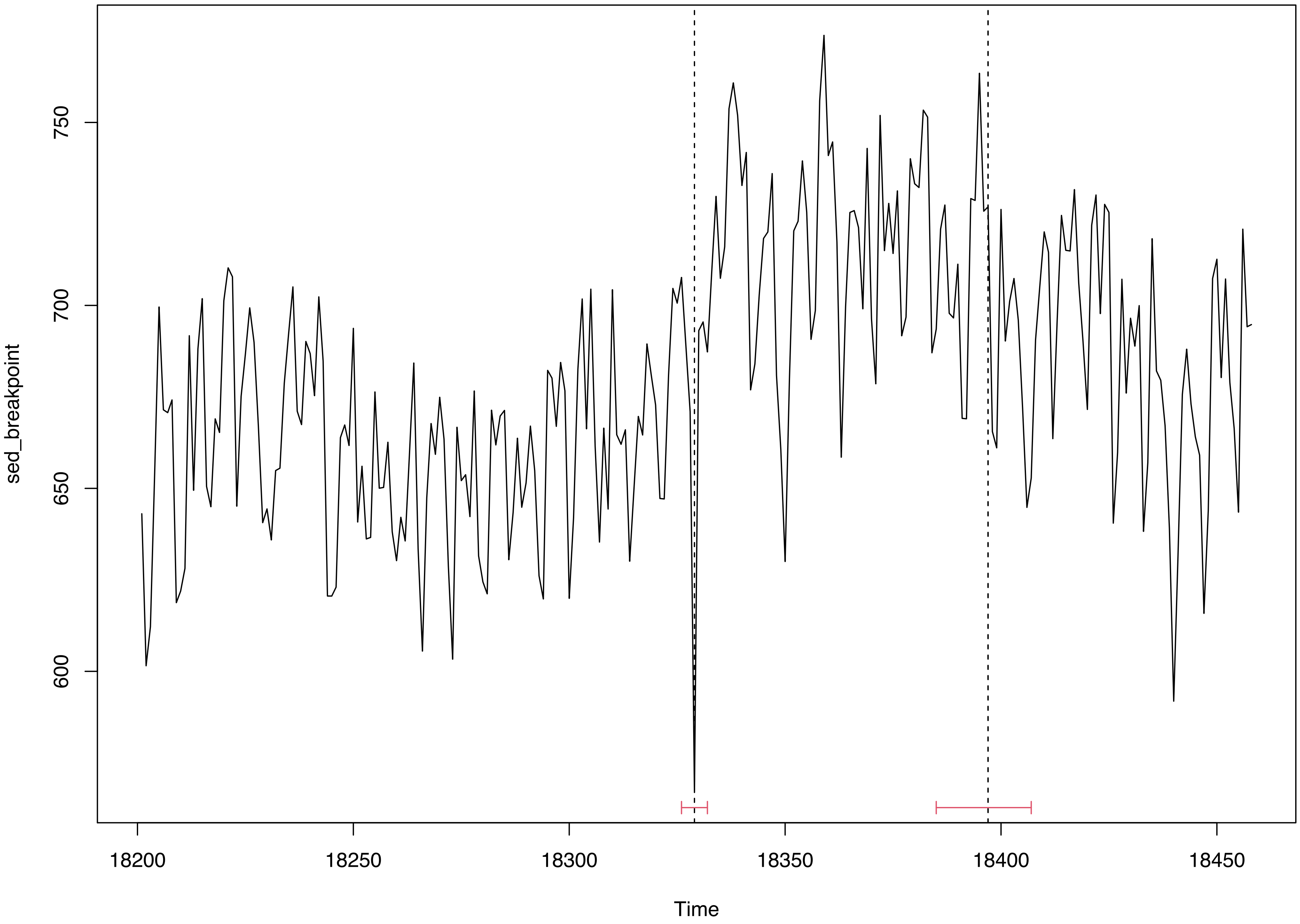

Supplement: Multimedia Appendix 1 [file publichealth_v7i11e28317_app1.pdf]
