# Supplementary material for: Impact of the COVID-19 Pandemic on Objectively Measured Physical Activity and Sedentary Behavior Among Overweight Young Adults: Yearlong Longitudinal Analysis
Source: JMIR Public Health Surveill. 2021 Nov 24;7(11):e28317. doi: 10.2196/28317 (PMC8614391; doi:10.2196/28317)
Supplement: Multimedia Appendix 2 [file publichealth_v7i11e28317_app2.pdf]

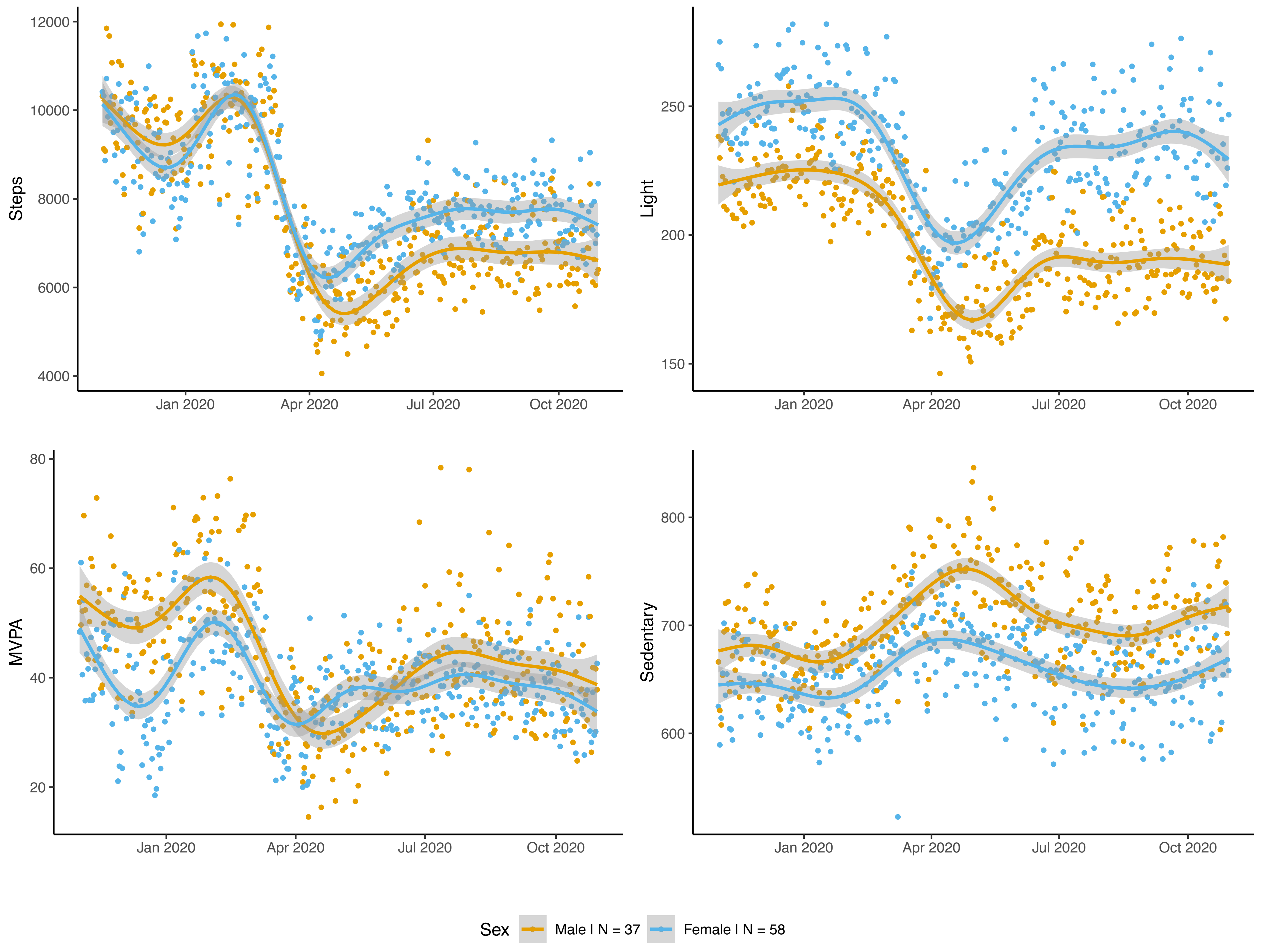
Steps
12000
10000
8000
6000
4000
Jan 2020
Apr 2020
Jul 2020
Oct 2020
Light
250
200
150
MVPA
80
60
40
20
Sedentary
800
700
600
Sex
Male I N = 37
Female I N = 58

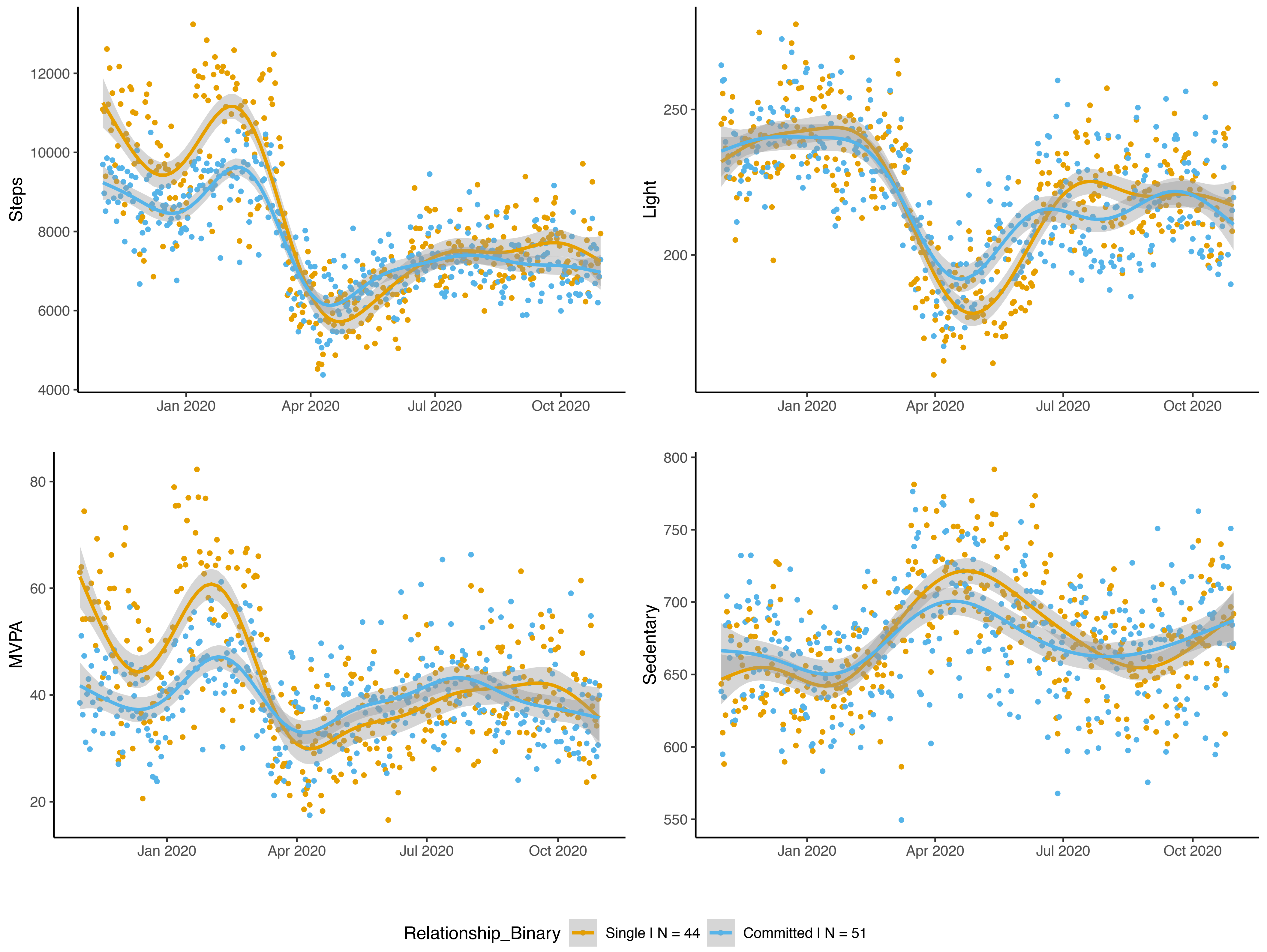

Steps
12000
10000
8000
6000
4000
Jan 2020
Apr 2020
Jul 2020
Oct 2020
Light
250
200
MVPA
80
60
40
20
Sedentary
800
750
700
650
600
550
Relationship_Binary
Single | N = 44
Committed | N = 51

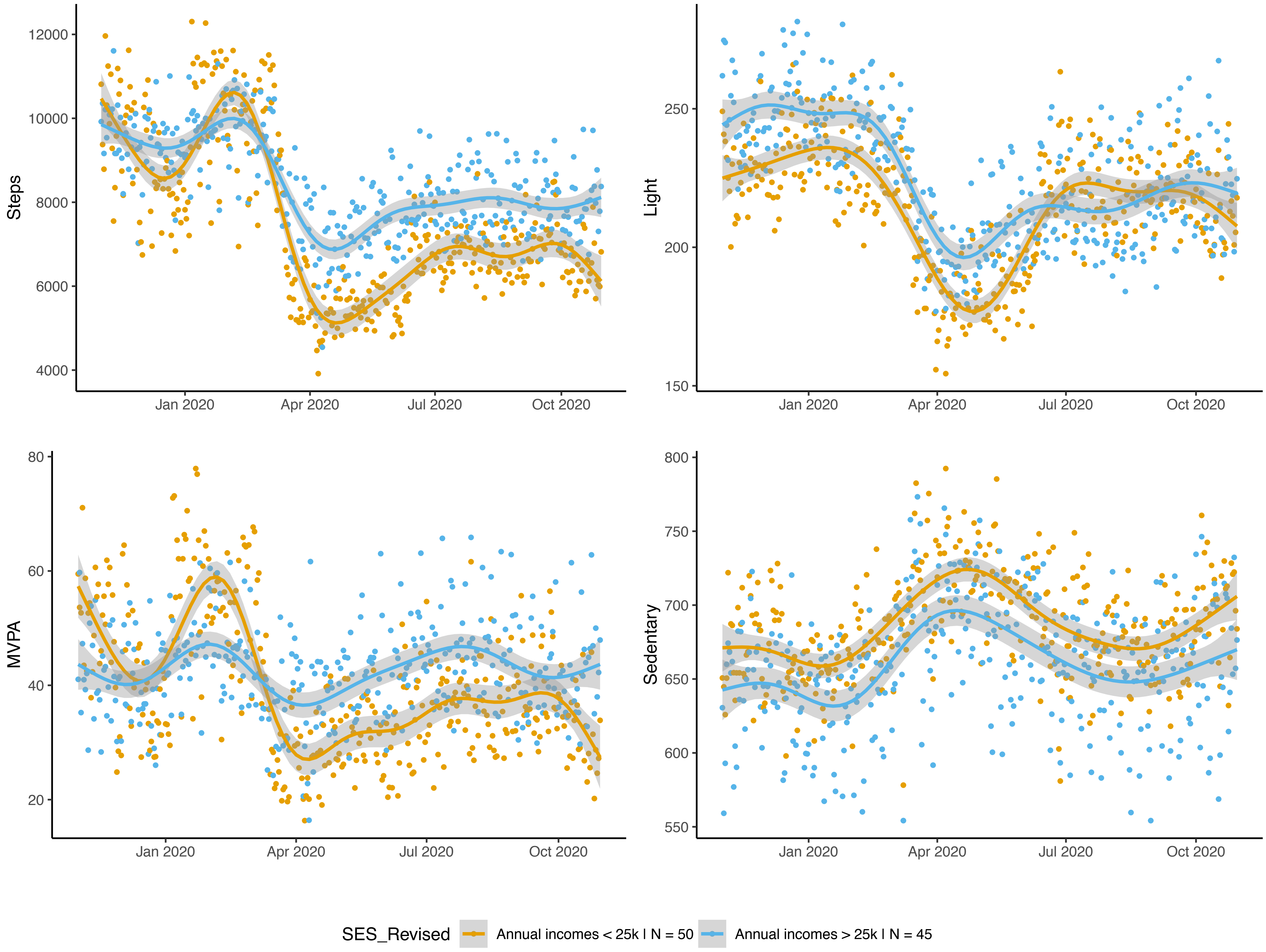

Steps
Light
MVPA
Sedentary
Jan 2020
Apr 2020
Jul 2020
Oct 2020
SES_Revised
Annual incomes < 25k | N = 50
Annual incomes > 25k | N = 45

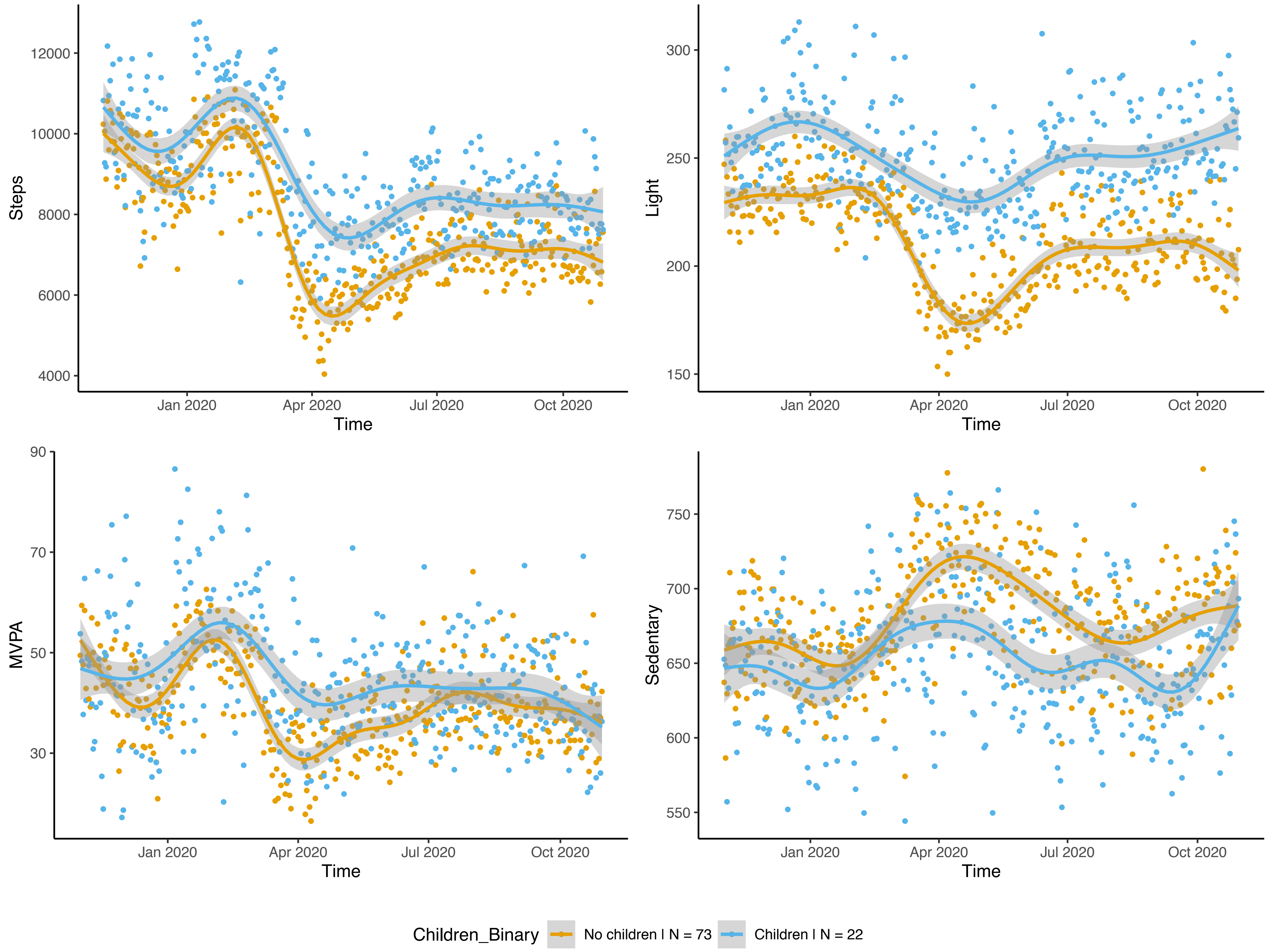
Steps
12000
10000
8000
6000
4000
Jan 2020
Apr 2020
Jul 2020
Oct 2020
Time
Light
300
250
200
150
MVPA
90
70
50
30
Sedentary
750
700
650
600
550
Children_Binary
No children I N = 73
Children I N = 22
